# Dietary Efficacy Evaluation by Applying a Prediction Model Using Clinical Fecal Microbiome Data of Colorectal Disease to a Controlled Animal Model from an Obesity Perspective

**DOI:** 10.3390/microorganisms10091833

**Published:** 2022-09-14

**Authors:** Hochan Seo, Cheol-O Kwon, Joo-Hyun Park, Chil-Sung Kang, Tae-Seop Shin, Eun-Young Yang, Jin Woo Jung, Byoung-Seok Moon, Yoon-Keun Kim

**Affiliations:** 1 R&D Center of MD Healthcare Inc., 9, World Cup Buk-ro 56-gil, Mapo-gu, Seoul 03923, Korea; 2 R&D Center, HK inno.N Corporation, 811, Deokpyeong-ro, Majang-myeon, Icheon-si, Gyeonggi-do 17389, Korea; 3 Kolmar Korea R&D Complex, HK Kolmar Holdings, 61, Heolleung-ro 8-gil, Seocho-gu, Seoul 06800, Korea

**Keywords:** obesity, inflammatory bowel disease, colorectal cancer, microbiome, 16S amplicon sequencing, gradient boosting machine, *Nypa fruticans*

## Abstract

Obesity associated with a Western diet such as a high-fat diet (HFD) is a known risk factor for inflammatory bowel disease (IBD) and colorectal cancer (CRC). In this study, we aimed to develop fecal microbiome data-based deep learning algorithms for the risk assessment of colorectal diseases. The effects of a HFD and a candidate food (*Nypa fruticans*, NF) on IBD and CRC risk reduction were also evaluated. Fecal microbiome data were obtained from 109 IBD patients, 111 CRC patients, and 395 healthy control (HC) subjects by 16S rDNA amplicon sequencing. IBD and CRC risk assessment prediction models were then constructed by deep learning algorithms. Dietary effects were evaluated based on fecal microbiome data from rats fed on a regular chow diet (RCD), HFD, and HFD plus ethanol extracts or water extracts of NF. There were significant differences in taxa when IBD and CRC were compared with HC. The diagnostic performance (area under curve, AUC) of the deep learning algorithm was 0.84 for IBD and 0.80 for CRC prediction. Based on the rat fecal microbiome data, IBD and CRC risks were increased in HFD-fed rats versus RCD-fed rats. Interestingly, in the HFD-induced obesity model, the IBD and CRC risk scores were significantly lowered by the administration of ethanol extracts of NF, but not by the administration of water extracts of NF. In conclusion, changes in the fecal microbiome of obesity by Western diet could be important risk factors for the development of IBD and CRC. The risk prediction model developed in this study could be used to evaluate dietary efficacy.

## 1. Introduction

Obesity has become a public problem as Westernized diets, including high-sucrose and high-fat diets, have become more common for decades. From 1980 to 2013, the proportion of overweight adults increased by 28% in developed countries and nearly 60% in developing countries. Obesity is an epidemic issue with no country reporting a decrease in prevalence during that period [1]. A Westernized diet that includes a high-sucrose, high-fat diet is known to accelerate obesity-related metabolic syndrome. In previous studies, overweight and obese people have been found to be more susceptible to chronic localized inflammation than those of normal weight [2,3]. In epidemiological studies, obesity has been associated with liver, gallbladder, and pancreatic cancers [4]. Adipose tissue in the body plays an important role in immune homeostasis. It is known to induce changes in intestinal immunity and influence the intestinal microflora, intestinal barrier function, and innate and adaptive immune cells residing in the gut [5]. Symbiotic microbes have also been found to be associated with obesity through their contributions to dietary energy digestion, chronic systemic inflammation, changes in intestinal microbial composition, and energy homeostasis [6].

Inflammatory bowel disease (IBD) is known to be an important risk determinant of developing colorectal cancer (CRC) [7]. IBD patients have been observed to have a defective epithelial barrier and increased intestinal permeability [8]. Indeed, studies on human subjects have shown that the gut microbiome is different in patients with IBD from that in healthy control subjects [9]. Metagenomics of gut microbiota in patients with IBD have also proven a role of the microbiome in the pathogenesis of IBD [10]. *Fusobacterium* species, *Pasturellaceae*, *Proteobacteria* (adherent invasive *E.* *coli*), *Ruminococcus gnavus*, and *Veillonellaceae* have been found to be increased in patients with IBD^11^. Recently, a growing number of studies have reported changes in the gut microbiota in CRC samples, suggesting that the gut microbiota may be an essential contributing factor in the initiation and development of this cancer [11]. As a result of studies of the microbial composition of colorectal cancer patients, pro-inflammatory pathogenic bacteria are increased, and intestinal homeostasis-preserving bacteria are decreased [12,13,14].

Human microbiome studies have been conducted to predict and understand host phenotype and microbiome composition. In previous studies, statistical methods such as the t-test, ANOVA, and Mann–Whitney U test have been used to simply find a significant difference in the increase or decrease of microbiome abundance [14,15,16,17]. Methods such as logistic regression have been used to classify the phenotype of the host. However, using these methods without testing the distribution or transformation of microbiome data has limitations. In addition, public databases providing taxonomy specify taxonomy assignment loosely at the genus level. Considering these limitations of statistical testing analysis and taxonomic assignment, we are interested in performing predictive analysis for microbiome data using machine learning AI algorithms. We employ machine learning methods to make predictions of a phenotype based on the taxonomic hierarchical values of microbiome data. The classification model developed based on previous studies has an area under the curve (AUC) of about 0.8, showing accuracy in areas such as the fecal occult blood test, a standard non-invasive clinical test for CRC, confirming its usefulness [18]. However, there are few cases where the usefulness of the classification model has been demonstrated by applying it to a controlled disease model.

*Nypa fruticans* (NF) Wurmb is a perennial plant. It is the sole species in the genus *Nypa*. It inhabits mostly mangrove areas on shorelines. It is distributed all over southeast Asia including India, Sri Lanka, and Myanmar. It is also distributed in Japan and Australia [19,20]. It is used for palm sugar and as an alcoholic beverage. Vinegar produced by the fermentation of the sap of nipa palm, in particular, is a traditional vinegar used as a folk remedy in Malaysian society to treat diabetes [21,22]. NF is also traditionally used for treating dental-related inflammation in Myanmar [23]. NF is rich in polyphenols and antioxidants, which are associated with anti-inflammatory properties [24]. Studies on the physiological activity of NF have also demonstrated antioxidant and antidiabetic efficacies of nipa palm vinegar [25]. In addition, the photochemical analysis of NF has demonstrated the antioxidant efficacy of seed mesocarp and sap [26,27] and the anti-hyperglycemic and antinociceptive activity of leaf and stem extracts [28].

In this study, the performance of classification for the host phenotype was evaluated by creating a risk assessment model using machine learning techniques based on the stool 16S rRNA metagenomic results of IBD, CRC patients, and healthy controls. In addition, by evaluating the performance of predicting disease risk in a rat model induced by a high-fat diet, an external performance assessment was conducted. This suggests that the risk assessment model using stool metagenome data is useful for predicting disease risk levels.

## 2. Materials and Methods

### 2.1. Subjects and Sample Collection

Fecal samples by group were collected from 395 healthy controls, 109 patients with IBD, and 111 patients with CRC in six hospitals. As for the healthy control group, 321, 36, and 38 samples were collected at Haeundae Paik Hospital, Seoul National University Bundang Hospital, and Boramae Medical Center, respectively. The healthy controls recruited in this study visited the hospital for regular medical checkups. After the examination, a healthy control group with normal test results without known diseases was selected. The criteria for excluding healthy controls were: diagnosis of colon disease, treatment, and past CRC diagnosis. For the IBD group samples, 69 samples of ulcerative colitis (UC) and 40 samples of Crohn’s disease (CD) were collected at Seoul National University Bundang Hospital. A total of 72, 11, 12, and 16 samples of CRC patients were collected from Seoul National University Bundang Hospital, Chung-Ang University Hospital, Ewha Womans University Mokdong Hospital, and Dankook University Hospital, respectively. This study protocol was approved by the Institutional Review Board of each hospital, including Haewoondae Baek Hospital (IRB No. 129792-2015-064), Seoul National University Bundang Hospital (IRB No. B-1708/412-301), Boramae Medical Center (IRB No. 20161020/16-2016-136/111), Chung-Ang University Hospital (IRB No. 1772-001-290), Ewha Womans University Mokdong Hospital (IRB No. 2017-09-012-003), and Dankook University Hospital (IRB No. 2017-10-004). All procedures in this study followed approved guidelines. Prior consent was obtained from all clinical trial subjects.

All participants ate a typical Korean diet rich in rice and fermented soy products, avoiding the consumption of products such as probiotics that could affect the analysis the day before stool collection. The participants did not smoke or drink alcohol. The samples did not touch water, as a kit was used. The stool samples were collected from the center of the stool using a spoon attached to the kit and were stored below –20 °C.

### 2.2. Experimental Design for High-Fat Diet Model in Rat

A total of 32 rats were divided into four groups: regular chow diet (Normal), high-fat diet (HFD), NF ethanol extract with HFD (NFE), and NF water extract with HFD (NFW). All groups were enrolled from Koatech (Pyeongtaek, Gyeonggi-do, Korea) from day 0 to day 42 every week with eight replications. The animal experiments were conducted at KNOTUS (Guri, Korea). To administer the medication, the rate was fixed by the cervical skin fixation method and administered directly into the stomach using a sonde for oral administration. The Normal groups were injected with a vehicle (10 mL/kg) without HFD. The HFD groups were injected with a vehicle (10 mL/kg) and HFD. The NFE groups were injected with NFE (500 mg/kg, 10 mL/kg) and HFD once a day for 6 weeks. The NFW groups were injected with NFW (500 mg/kg, 10 mL/kg) and HFD once a day. Stool samples were collected every week and stored in a cryogenic freezer (about –70 °C).

### 2.3. DNA Extraction and NGS Library Preparation for Sequencing of 16S rRNA Amplicon

DNA was extracted from each stool using a DNeasy PowerSoil kit (QIAGEN, Düsseldorf, Germany) according to the manufacturer’s instructions. The extracted DNA was quantified using a QIAxpert system (QIAGEN, Düsseldorf, Germany). The 16S rDNA was amplified using V3 forward primer (5′-TCGTCGGCAGCGTCAGATGTGTATAAGAGACAGCCTACGGGNGGCWGCAG-3′) and V4 reverse primer (5′-GTCTCGTGGGCTCGGAGATGTGTATAAGAGACAGGACTACHVGGGTATCTAATCC-3′). Index PCR with sequencing adapters attached to amplified DNA was performed using a Nextera XT Index kit (Illumina, San Diego, CA, USA). After quantifying, normalizing, and pooling the libraries, each amplicon was sequenced using a MiSeq instrument (Illumina, San Diego, CA, USA).

### 2.4. Microbiome Profiling

Paired-end reads that matched adapter sequences were trimmed with Cutadapt version 1.1.6 [29]. The resulting FASTQ files containing paired-end reads were merged with CASPER and then quality filtered with the Phred (Q) score-based criteria described by Bokulich [30,31]. Any reads shorter than 350 bp or longer than 550 bp after merging were also discarded. To identify chimeric sequences, a reference-based chimera detection step was conducted with VSEARCH against the SILVA gold database [32,33]. Next, sequence reads were clustered into operational taxonomic units (OTUs) using VSEARCH with a closed-reference clustering algorithm under a threshold of 97% sequence similarity. Representative sequences of OTUs were finally classified using the SILVA 132 database with UCLUST (parallel_assign_taxonomy_uclust.py script on QIIME version 1.9.1) under default parameters [34]. The Chao1 index, an estimator of the richness of taxa per individual, was estimated to measure the diversity of each sample. If clusters could not be assigned at the genus level due to a lack of sequences or redundant sequences in the database, the taxon was assigned at the next highest level as indicated in parentheses.

### 2.5. Data Preprocessing through Taxonomic Accumulation

Before using features for the model, we employed a novel hierarchal accumulation method to give weight to imprecisely classified genera through their verified upper-level taxonomy, while simultaneously reducing the influence of zero-inflation in the microbiome relative abundance data. First, the assigned species were summarized to their respective genus level and all abundance values were scaled by log2. Next, the sum of the taxa abundance values for each sample was set to one to represent the relative abundance of each OTU. Finally, taxonomic values from the genus to the kingdom level were accumulated from all samples using the following formula:

V_ACCUMULATION_ = V_G_ + (k_1_ × V_F_) + (k_2_ × V_O_) + (k_3_ × V_C_) + (k_4_ × V_P_) + (k_5_ × V_K_) (1)

V_ACCUMULATION_: accumulated value of a genus.

V_G_: relative abundance of a genus.

V_f/O/C/P/K_: sum of V_G_ for genus’ higher taxonomy (family, order, class, phylum, kingdom).

k_i_ = 10^−(1 + i)^.

The accumulated taxonomic values of the sequences obtained from bacteria in all samples were calculated as described above and used for the downstream disease risk predictive model.

### 2.6. Risk Assessment Model

After data preprocessing, 1639 taxa from CRC and 809 taxa from IBD were selected as features for the colon disease model development. We selected the GBM method as a disease classification model. The GradientBoostingRegressor function in the scikit-learn package (version 0.21.3) was used in Python [35]. To overcome limitations caused by overfitting, cross-validation was applied with data randomly split into training sets and test sets at a 7:3 ratio for 30 iterations. Training data sets were used for model training and test sets were used for validation.

### 2.7. Statistical Analysis

The age difference between the healthy control group and the patient group was tested using a t-test. The gender difference was tested using a chi-squared test. The alpha diversity of the microbial composition was measured using the Simpson diversity index and rarefied to compare between the patient group and healthy control group. It was rarefied to 1099, which is the minimum read depth of the total samples. The beta diversity was analyzed using principal coordinate analysis (PCoA) based on Bray–Curtis dissimilarity. Significant differences between the healthy control group and the patient group were determined using a t-test for continuous variables. Before using the t-test, an f-test was performed to ensure that the variances were equal. Additionally, the Kruskal–Wallis test and Mann–Whitney U test were performed to analyze the microbiome differences in rat stool samples, with the *p*-value corrected using the Benjamini–Hochberg method. Significant differences were considered if the *p*-value was less than 0.05 or the adjusted *p*-value (Ad. *p*) was less than 0.05. All statistical analyses were performed using R version 4.1.0 (Vienna, Austria).

## 3. Results

### 3.1. Clinical Characteristics of Subjects

To establish a risk assessment model based on the stool microbial profiles of the healthy control group and IBD and CRC patient groups, there was no statistically significant difference in age between the groups (*p* = 0.313 for healthy control group vs. IBD group, *p* = 0.462 for healthy control group vs. CRC group). However, there was a statistically significant difference in gender between the control group and the patient group (*p* < 0.001 for healthy control group vs. IBD group, *p* = 0.001 for healthy control group vs. CRC group). The mean age of the control group used in the IBD assessment model was 39.99 years. The mean age of IBD patients was 38.27 years. The mean age of the control group used in the CRC assessment model was 62.75 years. The mean age of CRC patients was 63.5 years, as shown in Table 1.

### 3.2. Comparison of Alpha and Beta Diversity between Healthy Controls and IBD Patients

**Figure 1:**
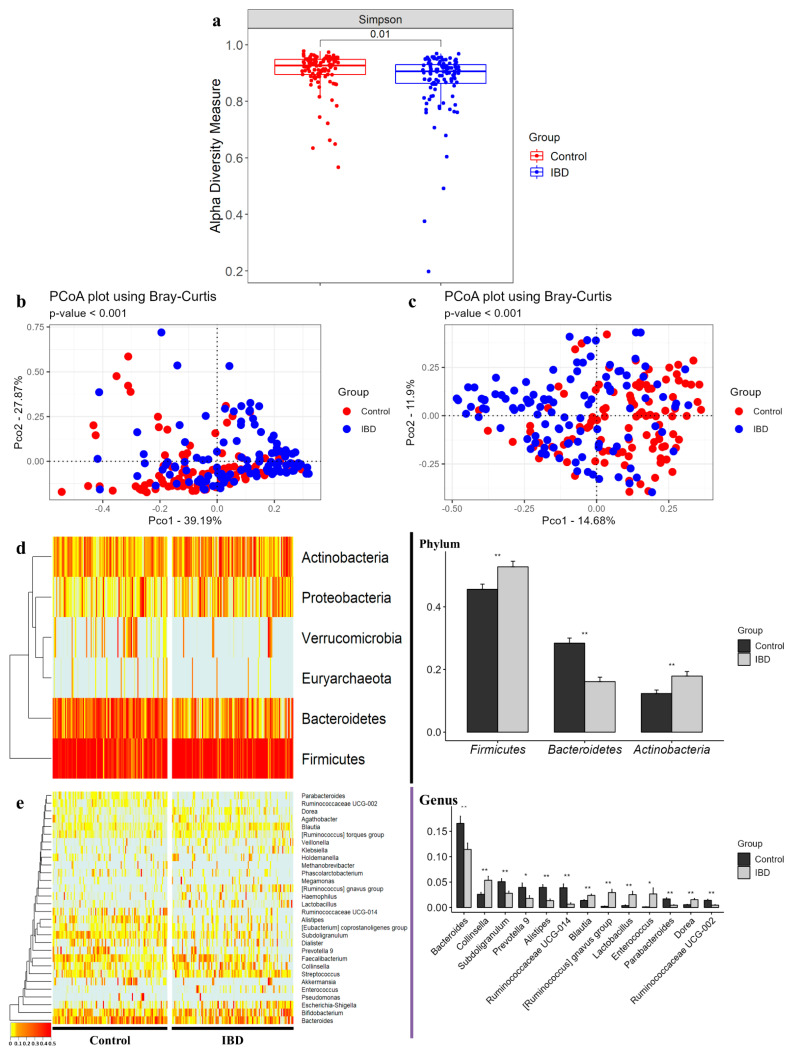
Alpha diversity, beta diversity, and compositions of microbiota at phylum and genus levels in IBD clinical samples. (**a**) Alpha diversity was defined by Simpson index. Beta diversity based on Bray-Curtis dissimilarity was measured at (**b)** phylum and (**c**) genus levels. The significance test of beta diversity was performed using PERMANOVA. Left-side heatmap plots and hierarchical clustering dendrograms show microbial compositions between individual samples of control and IBD groups at (**d**) phylum and (**e**) genus levels. Right-side bar plots highlight different average relative abundance of individual key taxa between the control and IBD subjects’ stool microbiota at (**d**) phylum and (**e**) genus levels. Significance between groups was assessed by a *t*-test (*, *p* < 0.05; **, *p* < 0.01).

The Simpson diversity index was used to calculate the alpha diversity of the microbial composition of the healthy control group and IBD patient group. The IBD patient group showed a significant decrease (*p* = 0.01) in the Simpson index compared to the healthy control group (Figure 1a). Beta diversity was measured based on PCoA using Bray–Curtis dissimilarity to determine the segregation of the clinical samples. The beta diversity significantly separated the clinical samples at both phylum and genus levels (*p* < 0.001) (Figure 1b,c).

### 3.3. Differences in Gut Microbiome Abundance between Healthy Controls and IBD Patients

At the phylum level, *Firmicutes* and *Actinobacteria* were significantly increased in the IBD group, whereas *Bacteroidetes* was significantly decreased (Figure 1d). At the genus level, *Bacteroides*, *Subdoligranulum*, *Alistipes*, *Ruminococcaceae* UCG-014, *Parabacteroides*, and *Ruminococcaceae* UCG-002 were significantly more decreased in the IBD group (*p* < 0.01), whereas *Collinsella*, *Blautia*, [*Ruminococcus*] gnavus group, *Lactobacillus*, and *Dorea* were significantly increased (*p* < 0.01) compared with the healthy control group. Notably, *Enterococcus*, [*Ruminococcus*] gnavus group, *Lactobacillus*, *Ruminococcaceae* UCG-014, *Parabacteroides*, *Ruminococcaceae* UCG-002, and *Alistipes* showed drastic fold changes (23-, 13-, 7-, 0.17-, 0.27-, 0.32-, and 0.34-fold, respectively) (Figure 1e) (Figure S1).

### 3.4. Comparison of Alpha and Beta Diversity between Healthy Controls and CRC Patients

**Figure 2:**
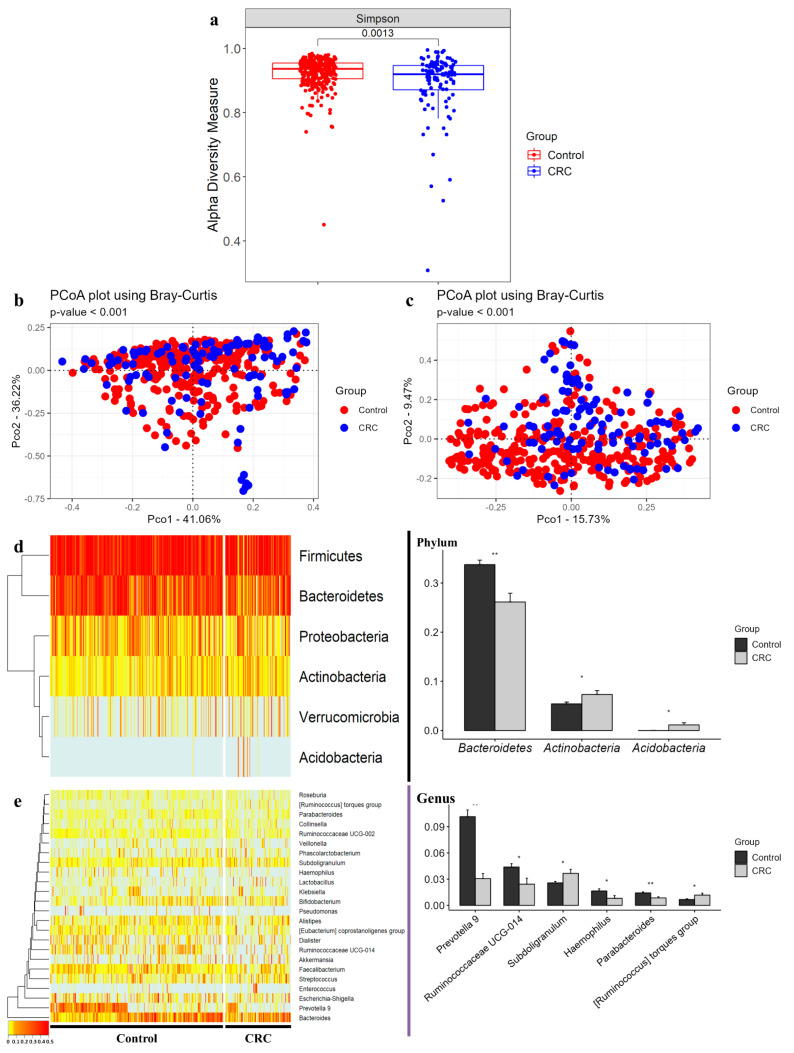
Alpha diversity, beta diversity, and compositions of microbiota at phylum and genus levels in CRC clinical samples. (**a**) Alpha diversity was defined by Simpson index. Beta diversity based on Bray-Curtis dissimilarity was measured at (**b**) phylum and (**c**) genus levels. Left-side heatmap plots and hierarchical clustering dendrograms show microbial compositions between individual samples of control and CRC groups at (**d**) phylum and (**e**) genus levels. Right-side bar plots highlight different average relative abundance of individual key taxa between the control and CRC subject stool microbiota at (**d**) phylum and (**e**) genus levels. Significance between groups was assessed by a t-test (*, *p* < 0.05; **, *p* < 0.01).

Alpha diversity of the microbial composition of the healthy control group and CRC patient group was measured using the Simpson diversity index. Through this analysis, the CRC group showed a significantly lower diversity index than the control group (*p* = 0.0013) (Figure 2a). Beta diversity was measured based on PCoA using Bray–Curtis dissimilarity to determine the segregation of the clinical samples. At both the phylum and genus levels, the clinical samples were separated significantly (*p* < 0.001) (Figure 2b,c).

### 3.5. Differences in Gut Microbiome Abundance between Healthy Controls and CRC Patients

As a result of metagenomic analysis at the phylum level, *Actinobacteria* and *Acidobacteria* were significantly higher in the case group than in the control group, while the carriage of *Bacteroidetes* was significantly lower in the CRC patient group than in the healthy control group (*p* < 0.05). *Bacteroidetes* and *Actinobacteria* showed fold changes of 0.77- and 1.35-fold, respectively (Figure 2d). Notably, *Acidobacteria* showed a 64-fold difference between the CRC patients and healthy subjects. At the genus level, *Subdoligranulum* and the [*Ruminococcus*] torques group were increased, whereas *Prevotella* 9, *Ruminococcaceae* UCG-014, *Haemophilus*, and *Parabacteroides* were significantly decreased (*p* < 0.05) in the CRC group compared with the control group. *Haemophilus*, *Ruminococcaceae* UCG-014, and *Parabacteroides* showed fold changes of 0.49-, 0.55-, and 0.59-fold, respectively. The [*Ruminococcus*] torques group, *Subdoligranulum*, and *Prevotella* 9 showed 1.7-, 1.4-, and 0.3-fold changes, respectively (Figure 2e) (Figure S2).

### 3.6. Establishment and Performances of IBD and CRC Risk Assessment Models

**Figure 3:**
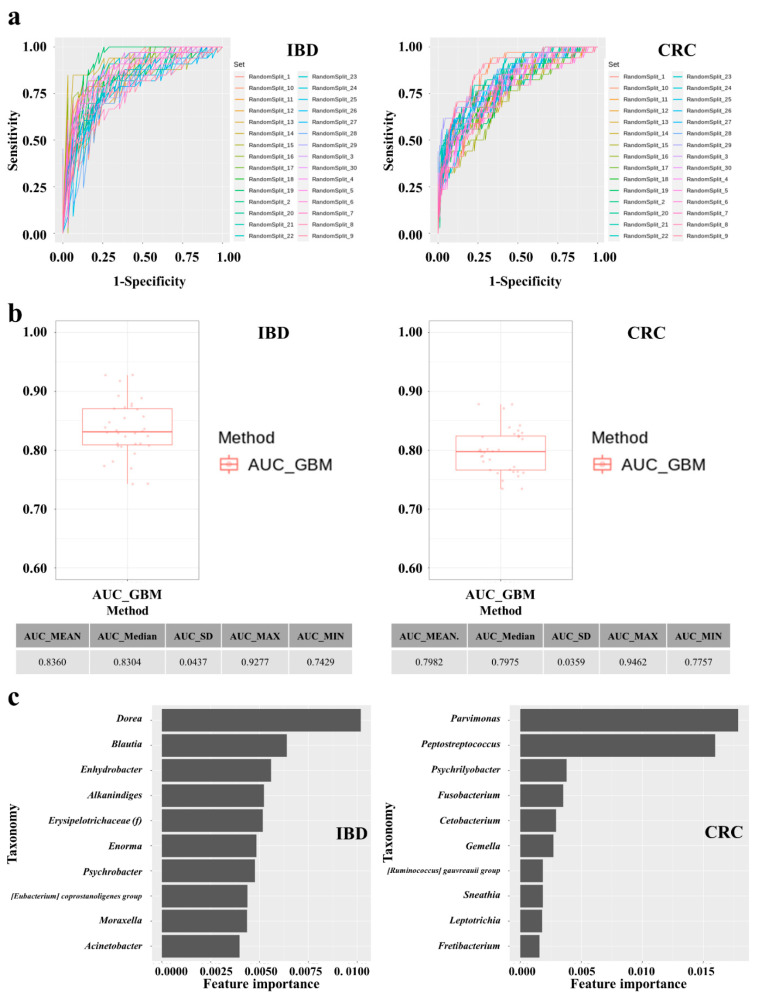
Performance of IBD and CRC risk predictive models. (**a**) ROC curves of 30 iterations of IBD and CRC risk prediction models developed by the GBM machine learning method. (**b**) AUC values for 30 iterations of IBD and CRC risk prediction models developed using the GBM machine learning method visualized in a box plot. (**c**) Feature importance of diagnostic models were assessed based on permutation feature importance analysis of stool microbial taxa.

Diagnostic model sets were developed to determine IBD and CRC risk by applying machine learning methods to the stool metagenomic data. The model performance was evaluated based on the resulting receiver operating characteristic (ROC) curve (Figure 3a). The AUC values from 30 iterations of the test were plotted as a box plot (Figure 3b). The disease classification performance of the IBD risk model was slightly better than that of the CRC risk model. In the IBD model, the mean AUC, standard deviation, minimum, and maximum of the model were 0.836, 0.0437, 0.7429, and 0.9277, respectively. In the CRC model, the mean AUC, standard deviation, minimum, and maximum of the model were 0.7982, 0.0359, 0.7757, and 0.9462, respectively (Figure 3b). The specificity and sensitivity of the set showing the highest AUC in the IBD model were 0.7419 and 0.9091, respectively. The specificity and sensitivity of the set showing the highest AUC in the CRC model were 0.8409 and 0.7059, respectively.

For the model with the highest ROC curve in each disease group model, we extracted the characteristic importance calculated as the mean and standard deviation of the accumulation of impurity reduction. As a result, in the model predicting the control group and IBD group, *Dorea*, *Blautia*, *Enhydrobacter*, and so on were shown as the genera to have a high importance in that order. In the model predicting the control group and CRC group, *Parvimonas*, *Peptostreptococcus*, *Psychrilyobacter*, and so on were shown as the genera with a high importance in that order (Figure 3c).

### 3.7. Body Weight Measure for 6 Weeks and Comparison of Alpha and Beta Diversity in HFD Induced Rat Model

**Figure 4:**
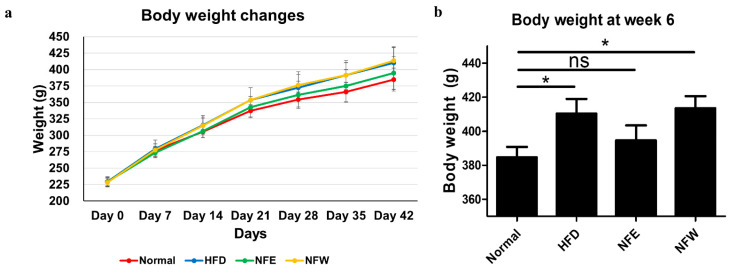
Body weight changes in the rat model induced by a high-fat diet. (**a**) Body weight changes over six weeks in four groups. (**b**) Body weight bar graph at week 6. The normal group had a regular chow diet. The HFD group had a high-fat diet. The NFE group had an ethanol extract with a high-fat diet. The NFW group had a water extract with a high-fat diet (*, *p* < 0.05; ns, not significant).

The body weight of the HFD model was measured to evaluate dietary efficacy of NF extracts for weight reduction. At 6 weeks, the body weight of the HFD group was higher than that of the normal group. The NFW group showed no difference in body weight compared to the HFD group, while the NFE group showed lower body weight than the HFD group (Figure 4a). As a result of expressing as a bar graph to compare the weight at 6 weeks of dietary intake, the normal group and the high-fat diet group showed a significant difference. However, there was no significant difference in weight between the NFE group and the normal group (Figure 4b).

**Figure 5:**
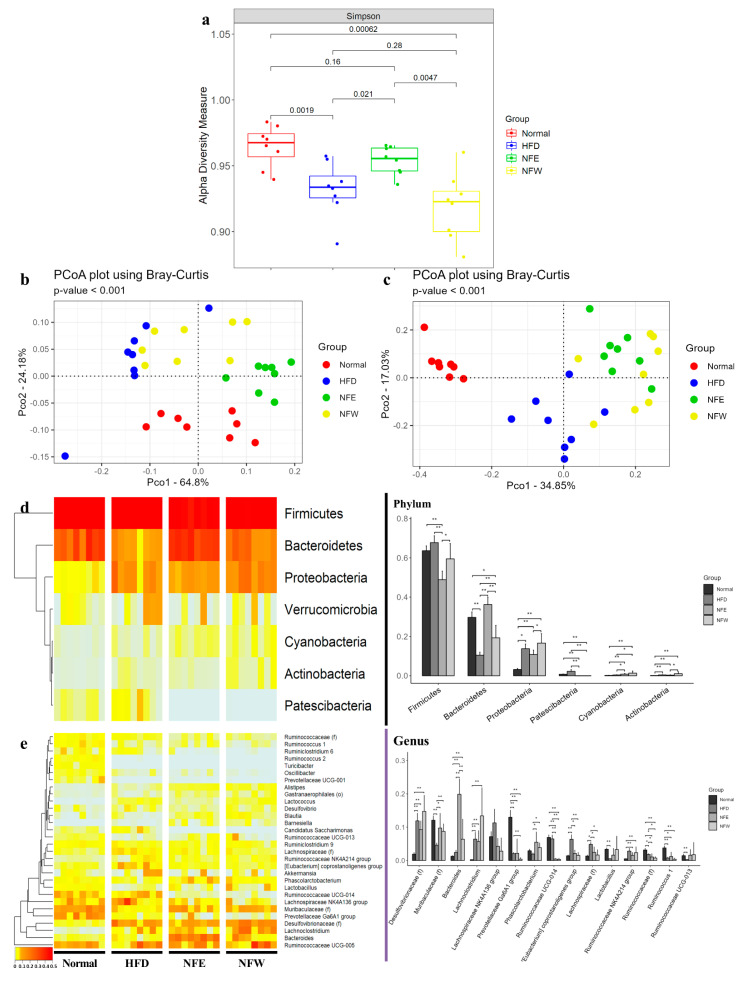
Alpha diversity, beta diversity, and compositions of microbiota at phylum and genus levels in rat stool samples. (**a**) Alpha diversity defined by observed OTUs, Chao1, and Shannon and Simpson indices. Beta diversity based on Bray-Curtis dissimilarity measure at (**b**) phylum and (**c**) genus levels. The left-side heatmap and hierarchical clustering dendrograms show microbial compositions between individual samples of normal, HFD, NFE, and NFW groups at (**d**) phylum and (**e**) genus levels. Right-side bar plots highlight different average relative abundance of individual key taxa subject stool microbiota at (**d**) phylum and (**e**) genus levels. Significance between groups was assessed by Mann–Whitney U test (*, Ad. *p* < 0.05; **, Ad. *p* < 0.01).

The alpha diversity of the gut microbiota composition in a murine model was measured using the Simpson index. As a result, the HFD group showed significantly lower enrichment than the normal group (*p* = 0.0019). The NFE group showed a significantly higher diversity value in the Simpson index than the HFD group (*p* = 0.021). The NFW group showed a significantly lower diversity value than the normal group (*p* = 0.0006), and was lower than the HFD group, although the difference was not statistically significant (*p* = 0.26) (Figure 5a). The beta diversity was measured based on PCoA plot using Bray–Curtis dissimilarity at the phylum and genus levels. At all levels, the rat stool samples of the four groups showed significant separation (*p* < 0.001) (Figure 5b,c).

### 3.8. Differences in Microbial Composition in HFD Induced Rat Model

We confirmed the microbiome configuration of the phylum level and the genus level and compared the difference among groups. At the phylum level, *Proteobacteria* and *Actinobacteria* were significantly higher in the HFD group than in the normal group, while *Bacteroidetes* was significantly lower in the HFD group than in the normal group. *Firmicutes* was significantly decreased in the NFE group than in the HFD group. The carriage of *Bacteroidetes* and *Cyanobacteria* was significantly increased in the NFE and NFW groups. However, *Patescibacteria* was significantly decreased in the two groups (Ad. *p* < 0.05) (Figure 5d). At the genus level, [*Desulfovibrionaceae*], *Lachnoclostridium*, [*Eubacterium*] coprostanoligenes group, and [*Lachnospiraceae*] were significantly higher in the HFD group than in the normal group, while [*Muribaculaceae*], *Prevotellaceae* Ga6A1 group, *Lactobacillus*, [*Ruminococcaceae*], *Ruminococcus* 1, and *Ruminococcaceae* UCG-013 were significantly lower in the HFD group. *Bacteroides* was significantly decreased in the NFE group than in the HFD group. The *Prevotellaceae* Ga6A1 group was significantly increased in the NFW group than in the HFD group. The carriage of [Muribaculaceae] and *Phascolarctobacterium* was significantly increased in NFE and NFW groups. However, *Ruminococcaceae* UCG-014, [*Eubacterium*] coprostanoligenes group, [*Lachnospiraceae*], and [*Ruminococcaceae*] were significantly decreased in both groups (Ad. *p* < 0.05) (Figure 5e) (Figure S3).

### 3.9. External Validation of Risk Assessment Models

**Figure 6:**
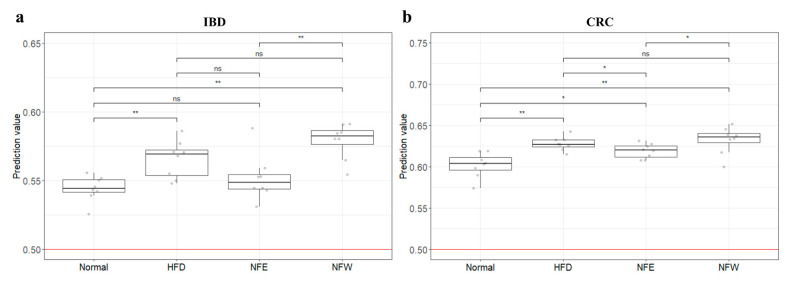
Risk prediction values of IBD and CRC models for rat groups. Predicted values of IBD (**a**) and CRC (**b**) risk were plotted as boxplots for normal, HFD, NFE, and NFW rat groups (*, *p* < 0.05; **, *p* < 0.01).

To determine whether the colorectal disease risk assessment model could evaluate well in a rigorous animal model, we calculated predictive values by substituting the rat stool metagenome data from the normal diet, HFD, NFE, and NFW groups. In both models for evaluating IBD and CRC risk, the predicted values in all groups were higher than 0.5, the standard for distinguishing the healthy control group from the patient group. However, the predicted risk score was increased in the HFD group compared with the normal diet group (Figure 6a,b). Among the groups taking NF with HFD, the NFW group tended to show increased risk scores while the NFE group tended to show a level lower than or similar to the normal group. This trend was more conspicuously observed in the IBD model than in the CRC model.

## 4. Discussion

In the present study, we constructed a deep learning-based risk prediction model using fecal microbiome data from IBD and CRC patients. As shown in Figure 3b, the AUC value used as predictive performance of the deep learning algorithm of the model was 0.84 for IBD and 0.80 for CRC. Although the microbial profiles from normal individuals and patients with IBD are sometimes obtained and compared, this comparison is seldom performed to examine the performances of these prediction models in controlled animal models [18,36,37]. We further evaluated the risk by applying this predictive model for colonic disease to a controlled obese rat model. As shown in Figure 6, the risk prediction model for IBD and CRC showed that the HFD group increased the risk compared to the regular diet group in the rat experiment. This is an important finding because it suggests that microbiome compositions induced by a high-fat diet and those of IBD and CRC are shared. Additionally, we assessed whether the consumption of NF extract reduced the risk of CRC or IBD when consumed during a high-fat diet. Our results confirmed the correlation that the intake of ethanol-extracted NFs could increase the microbiome composition, which lowers the IBD risk score. The novel approach suggests that disease risk prediction models can be established using well-controlled animal models and used as tools to assess dietary efficacy through changes in microbiome composition.

IBD is known to decrease the diversity of intestinal bacteria in the human gut [38,39]. According to previous studies, IBD patients have increased levels of *Proteobacteria*, *Pasteurellaceae*, *Veillonellaceae*, *Fusobacterium* species, and *Ruminococcus*, but decreased *Clostridium* groups IV and XIV, *Bacteroides*, *Suterella*, *Roseburia*, *Bifidobacterium* species, and *Fusobacterium prausnitzii* compared to healthy people [40,41]. In microbiome studies on CRC, it is known that relative abundances of *Faecalibacterium prausnitzii*, *Akkermansia muciniphila*, *Eubacterium rectale*, and *Ruminococcus bromii* are increased [42].

There are many reports on the relationship between food and gut microbiome changes or the association between microbiome composition changes and disease. The MetaHIT Consortium extracted DNA from human feces and compared a group with a low number of gut microbiome genes and a group with a high number of gut microbiome genes to calculate a low gut microbiome gene [43]. In the treated group, high inflammatory cytokine secretion and high obesity rates were confirmed. In addition, it was confirmed that the genes of intestinal microbes were increased in both groups through a caloric control diet for 6 weeks. It was also confirmed that increasing fiber through the intake of fruits and vegetables could increase the abundance of high symbiotic microorganisms [44]. In another study, as a result of comparing a low-gluten diet group with a normal diet group, the relative abundance of *Bifidobacterium* species was decreased in the group that adhered to a low-gluten diet. Furthermore, a decreased amount of *Bifidobacterium* species induces a reduction of IL-1β, which is produced by immune cell-induced inflammation, inflammasome, and cytokines [45]. With respect to cardiac metabolism, the symbiotic microorganism *Prevotella copri* has been shown to have a protective association between the Mediterranean dietary pattern and cardiometabolic health. Moreover, among animal models, a high-fat diet habit could delay the disease according to immune regulation by changing the intestinal microflora in a mouse auto-inflammatory bone disease model [46]. These results suggest that it is possible to change intestinal symbiotic microorganisms through dietary intervention for disease prevention and treatment. Furthermore, disease occurrence could be prevented by changing microbiome compositions.

In this study, we used a machine learning classification method to build a risk assessment model considering the characteristics of stool metagenomic data. With the development of NGS technology, research on the microbial metagenome has exploded over the past decade, producing a huge amount of data. Various classification models for data analysis have been developed. As a result, a machine learning algorithm has been adopted as a powerful tool for interpreting the complex big data output of microbiome profiling [47,48]. To overcome some general challenges of microbiome analysis, a new data preprocessing method was also developed and applied. Microbiome count data are generally very rich in zero values, leading to bias. Thus, such data need to be modified before they are used as model input features [49]. Additionally, taxonomic assignment is based on a public database in which many entries are loosely specified at the genus level [50]. Instead of eliminating all incorrectly labeled classifications to reduce data resolution, we used a new method of clustering taxonomic hierarchical values of a given OTU into a single code value. Through this method, we aimed to address these biases and improve the rationale for our risk prediction model. Although further verification is required, the high predictive strength shown through multiple iterations of the model trained with coded metagenomic data supports the advantages of the taxonomic hierarchical coding method.

Widely distributed from tropical to subtropical regions of southeast Asia and Oceania, *Nypa fruticans* Wurmb (Arecaceae), a palm tree, is traditionally used in Myanmar to treat dental-related inflammation [24]. In the stool microbial profile of the HFD-induced rat model, *Bacteroidetes*, *Phascolarctobacterium*, and *Lactobacillus* were increased at the genus level compared to the control group, while *Lachnospiraceae*, *Ruminococcaceae*, and UCG-014 [*Eubacterium*] were decreased. Ethanol-extracted NFs can lower the imbalance of these bacteria [51]. In particular, the increase in Bacteroidetes and Lactobacillus strains associated with obesity and lack of a significant difference between the normal diet group and the NFE group suggest that NFE may inhibit weight gain during HFD intake.

Our study has some limitations. A risk assessment model was created based on a human-derived fecal microbiome profile. However, its risk assessment was applied to a rat animal model. In addition, controlled clinical samples are extremely rare, making it an inevitable choice. Furthermore, although there are various models of colitis (UC/CD) in murine, these methods are mainly postulated to chemically alter the microbial profile by disrupting the mucosal barrier and inducing inflammation of the colon via intestinal permeation by symbiotic microbiota [52]. On the other hand, the HFD rat model, which can induce chronic inflammation by natural intestinal flora, was selected as a controlled external validation model for our colorectal cancer and colitis risk assessment model [53,54]. When the four groups were scored with the IBD and CRC risk assessment models based on identical fecal microbiome composition data from obese rats, the IBD model distinguished each group better than the CRC model. This leads us to infer that the microbiome induced by the HFD model is relatively correlated with IBD. However, in the case of CRC, the obesity model appears to be an unsuitable model for risk assessment. It is judged that the level of evaluation is not dramatic because cancer develops through a long process. If possible, controlled clinical samples would be more appropriate for risk assessment.

In conclusion, this study demonstrates that the risk prediction model generated based on the microbial profile of clinical disease can be externally validated by utilizing appropriate animal disease models. This study proves that a well-assessed microbiome profile-based prediction model can be a platform for rapidly estimating various indicators or markers or the efficacy of diets and foods.

## Figures and Tables

**Table 1:** Clinical characteristics of each disease group.

Disease Type	Age and Sex	Control	Case	*p*-Value
CRC	Age (mean) Sex (M:F)	62.75 292 (106:186)	63.5 111(61:50)	0.462 0.001
IBD	Age (mean) Sex (M:F)	39.99 103 (30:73)	38.27 109(82:27)	0.313 <0.001

M, male; F, female.

## Data Availability

The basic functions of the R language was used for risk assessment modeling and data preprocessing, and the GBM function of the scikit-learn package of Python was used for learning modeling. The source code for data preprocessing through taxonomic accumulation and parametric optimization for the Gradient Boosting Regressor function is available upon request.

## Supplementary Materials

The following supporting information can be downloaded at: https://www.mdpi.com/article/10.3390/microorganisms10091833/s1, Figure S1: Beta diversity and compositions of microbiota at class, order, family, and species levels in IBD clinical samples; Figure S2: Beta diversity and compositions of microbiota at class, order, family, and species levels in CRC clinical samples; Figure S3: Beta diversity and compositions of microbiota at class, order, family, and species levels in clinical samples.
