# Supplementary material for: Dietary Efficacy Evaluation by Applying a Prediction Model Using Clinical Fecal Microbiome Data of Colorectal Disease to a Controlled Animal Model from an Obesity Perspective
Source: Microorganisms. 2022 Sep 14;10(9):1833. doi: 10.3390/microorganisms10091833 (PMC9505706; doi:10.3390/microorganisms10091833)

Supplementary Information

Analysis at other ranks in every clinical samples

Supplementary Figure Legends

Supplementary Figure S1. Beta diversity and Composition of microbiota at class, order, family and species in IBD clinical samples

The left-side beta diversity analysis was visualized by the principal coordinate analysis(PCoA) method using the Bray-Curtis dissimilarity measure at the **(a)** class, **(b)** order and **(c)** family levels. The middle-side heatmap and clustering of individual control and IBD samples indicate relative abundance at the **(a)** class, **(b)** order and **(c)** family levels. Hierarchical clustering was measured by Euclidean distance. The right-side bar plots show average relative abundance of individual key taxa between control and IBD stool microbiota at the **(a)** class, **(b)** order and **(c)** family levels, with error bars representing the standard error (SE). Significant differences were calculated by a t-test (* = $p < 0.05$, ** = $p < 0.01$).

Supplementary Figure S2. Beta diversity and Composition of microbiota at class, order, family and species in CRC clinical samples

The left-side beta diversity analysis was visualized by the principal coordinate analysis(PCoA) method using the Bray-Curtis dissimilarity measure at the **(a)** class, **(b)** order and **(c)** family levels. The middle-side heatmap and clustering of individual control and CRC samples indicate relative abundance at the **(a)** class, **(b)** order and **(c)** family levels. Hierarchical clustering was measured by Euclidean distance. The right-side bar plots show average relative abundance of individual key taxa between control and CRC stool microbiota at the **(a)** class, **(b)** order and **(c)** family levels, with error bars representing the standard error (SE). Significant differences were calculated by a t-test (* = $p < 0.05$, ** = $p < 0.01$)

Supplementary Figure S3. Beta diversity and Composition of microbiota at class, order, family and species in clinical samples

The left-side beta diversity analysis was visualized by the principal coordinate analysis(PCoA) method using the Bray-Curtis dissimilarity measure at the **(a)** class, **(b)** order and **(c)** family levels. The middle-side heatmap and clustering of individual samples of normal, HFD, NFE and NFW groups indicate relative abundance at the **(a)** class, **(b)** order and **(c)** family levels. Hierarchical clustering was measured by Euclidean distance. The right-side bar plots show average relative abundance of individual key taxa between the normal, HFD, NFE and NFW rat stool microbiota at the **(a)** class, **(b)** order and **(c)** family levels, with error bars representing the standard error (SE). Significance between groups was assessed by Mann-Whitney U test (* = Ad. $p < 0.05$, ** = Ad. $p < 0.01$).

Supplementary Figure S1.

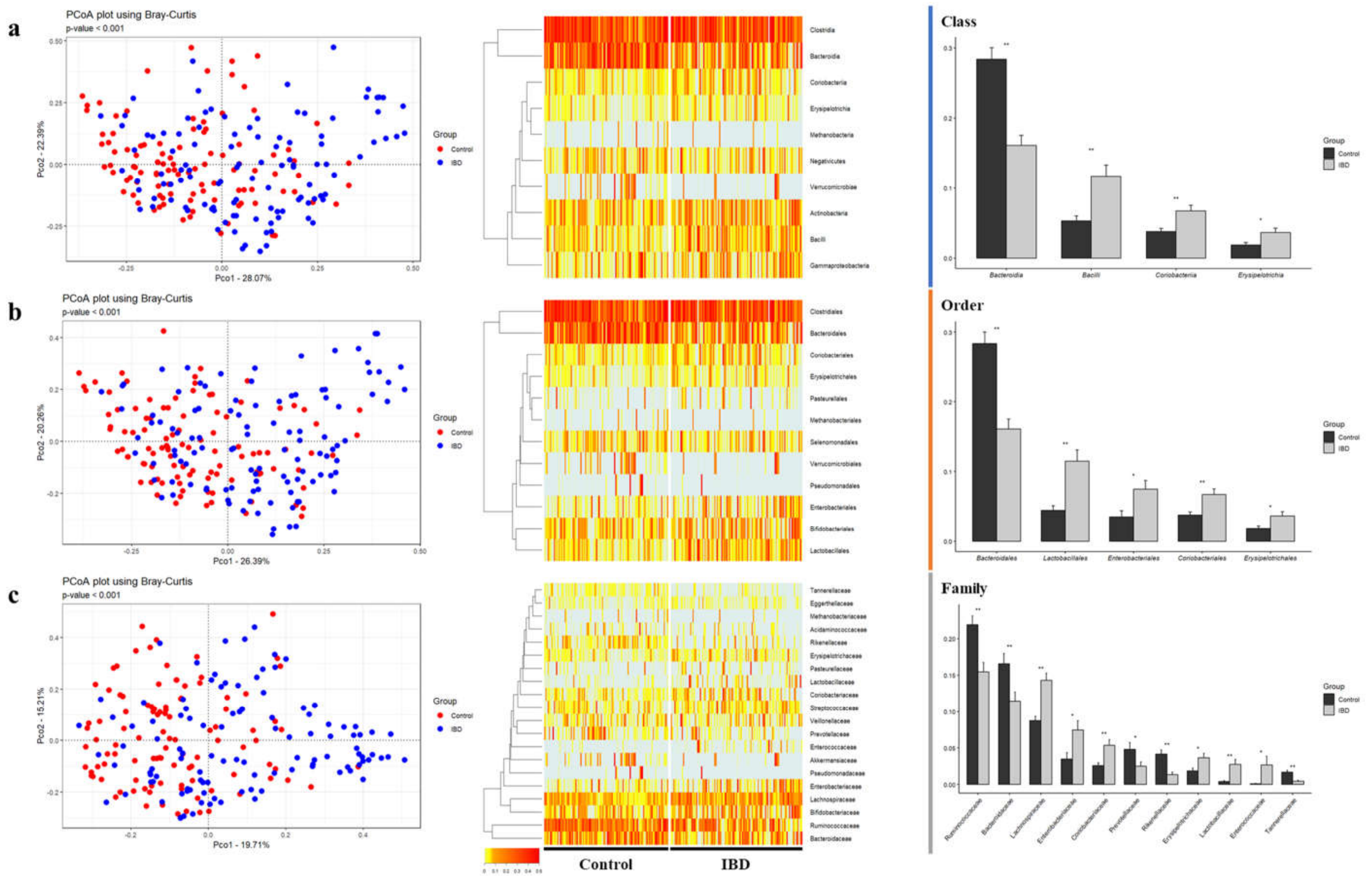

Supplementary Figure S2.

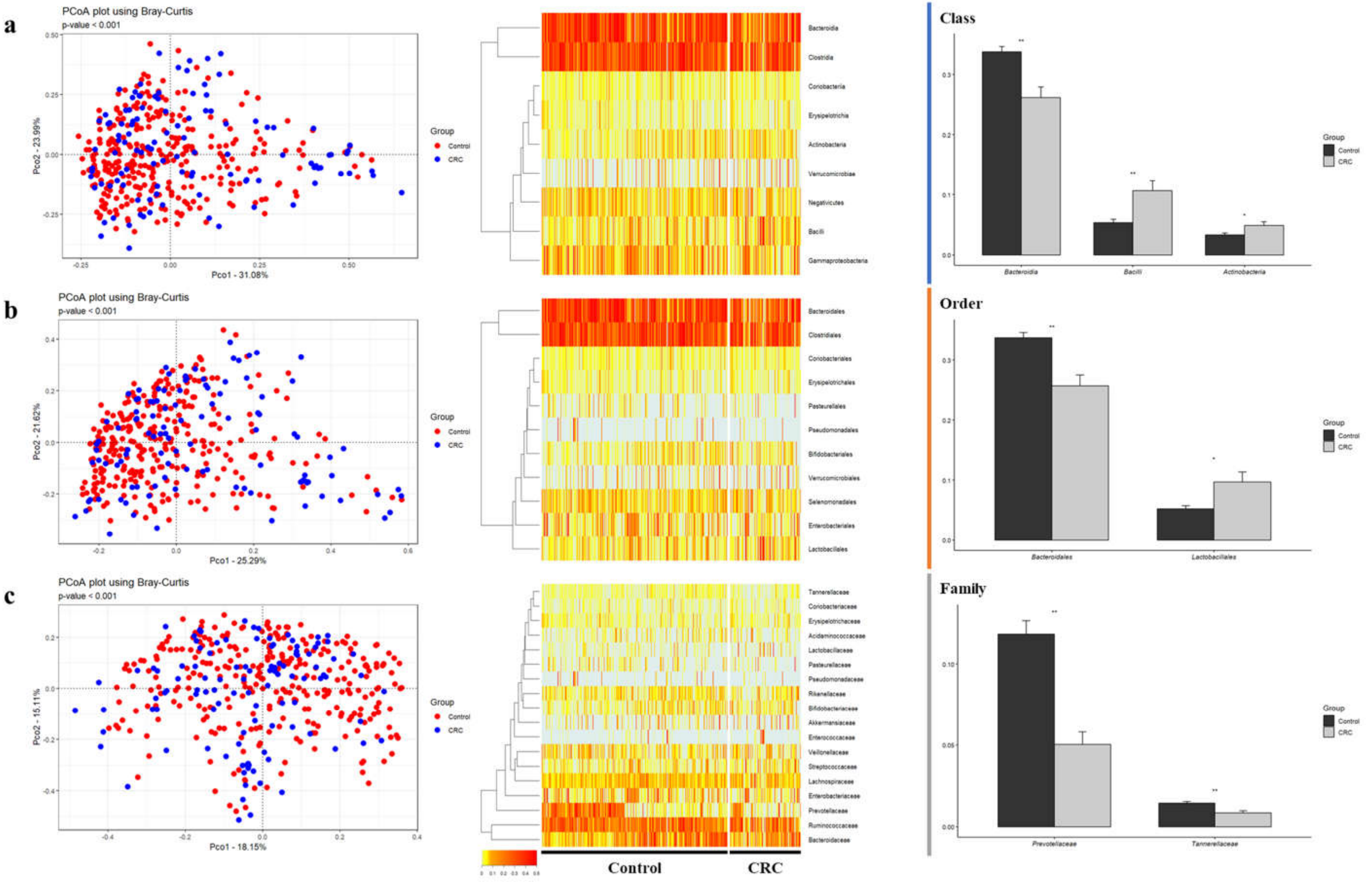

Supplementary Figure S3.

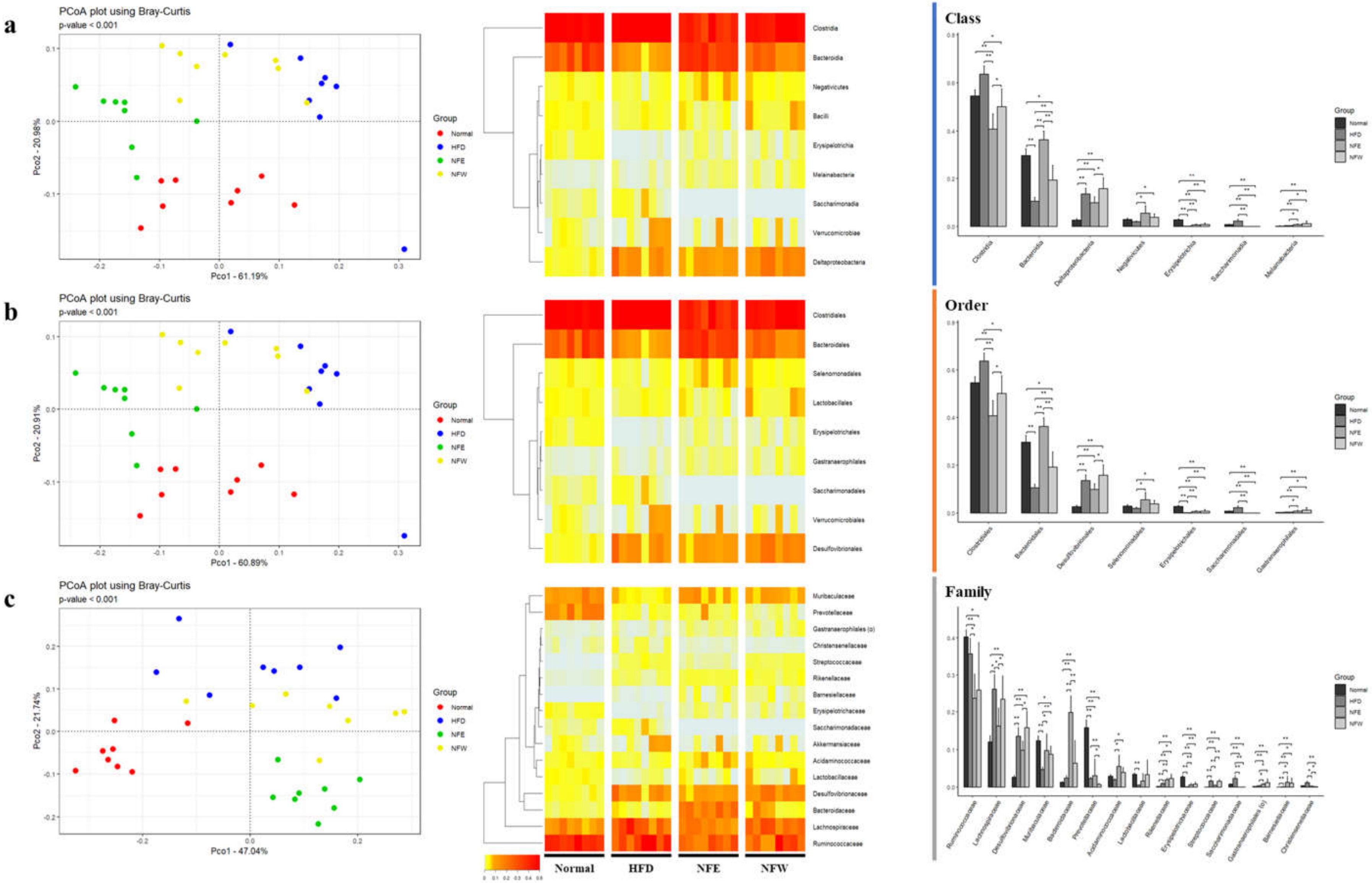

Supplement: Supplementary file 1 [file microorganisms-10-01833-s001.zip › microorganisms-1895333-supplementary.pdf]
